# Supplementary material for: High-resolution and high-accuracy topographic and transcriptional maps of the nucleosome barrier
Source: eLife. 2019 Jul 31;8:e48281. doi: 10.7554/eLife.48281 (PMC6744274; doi:10.7554/eLife.48281)
Supplement: Supplementary file 1. [file elife-48281-supp1.docx]

**Supplementary File 1**

**Oligos used in this study**

| Oligo | Sequence | Purpose |
| --- | --- | --- |
| ZC01 | 5’ATGTATGAGGTCTCGACCGCGGGAGTGATTTCCGTCTTACGGT 3’ | Left arm of Y |
| ZC02 | 5’phos-TCCTGAATGTCCGCGTCAGTAGTCAAAA-idsp-GACATTGTAAAGGCGGCGAGC 3’ |  |
| ZC03 | 5’biotin-CGGGAGTGATTTCCGTCTTACGGT 3’ | Right arm of Y |
| ZC04 | 3’CTTACAGGCGCAGTCATCAGTTTT-5’-5’ GACATTGTAAAGGCGGCGAGC 3’ |  |
| ZC05 | 5’ATGTATGAGGTCTCGAGGAGATGGACCCTATACGCGGCC 3’ | Alignment NPS for unzipping |
| ZC06 | 5’ATGTATGAGGTCTCGGTTGCAAGGTCGCTGTTCAATACATGCAC 3’ |  |
| ZC07 | 5’ATGTATGAGGTCTCGCAACGATGGACCCTATACGCGGCC 3’ | Loading NPS for unzipping |
| ZC08 | 5’ATACACAGGGTGCAAGGTCGCTGTTCAATACATGCAC 3’ |  |
| ZC09 | 5’phos-ccactctagaAAAGtctagagtggAGG 3’ | End hairpin |
| ZC10 | 5’NH_2_-TTAATTCATTGCGTTCTGTACACG 3' | 5’-CGGT oligo beads |
| ZC11 | 5’phos-CGGTCGTGTACAGAACGCAATGAATT 3’ |  |
| ZC12 | 5’tagataCGGCCGCAACggagaccTCATCACCATCATCCTGACTAGAGTCCTTGGCGAACC 3’ | pGMZ-8×repeat-2×BsaI |
| ZC13 | 5’cagtgctgcaatgataccgcgTgaGccacgctcaccggctccaga 3’ |  |
| ZC14 | 5’tctggagccggtgagcgtggCtcAcgcggtatcattgcagcactg 3’ |  |
| ZC15 | 5’tagtataGCTCTTCCGCTACTGcgagaccttcctcgctcactgactcgctgcgctcggtc 3’ |  |
| ZC16 | 5’AATTCATTGCGTTCTGTACACCAGG 3’ | Cross-linked DNA |
| ZC17 | 5’phos-GGTGTACAGAACGCAATGAATT 3’ |  |
| ZC18 | 5’ATGTATGAGGTCTCGCAACGATGGACCCTATACGCGGCC 3’ | NPS for Pol II assay |
| ZC19 | 5’ACATAGTATACACAGGGTGcgacggccagtgaattgtaatacg 3’ |  |
| ZC20 | 5’AGTATGTAGGTCTCG-CTTCGAGAAAATCAGCCAGCAGCG 3’ | 2 kb spacer DNA |
| ZC21 | 5’ATGTATGAGGTCTCG-ACCGGTGTTGGCCAGCATGATACG 3’ |  |
| ZC22 | 5’biotin-TTGCGCCACGGACAGTATGTC 3’ | 1.5 kb  biotin handle |
| ZC23 | 5’ATGTATGAGGTCTCG-ACCGGTGTTGGCCAGCATGATACG 3’ |  |
| ZC24 | 5’ATGTATGAGGTCTCGcaacGCCTCCCGGGCTcaccatcatcct 3’ | 1×repeat template |
| ZC25 | 5’ATGTATGAGGTCTCGAGGACGCAGCGGTGCGGAATAGGTCACG 3’ |  |
