# Supplementary material for: High-resolution and high-accuracy topographic and transcriptional maps of the nucleosome barrier
Source: eLife. 2019 Jul 31;8:e48281. doi: 10.7554/eLife.48281 (PMC6744274; doi:10.7554/eLife.48281)
Supplement: Supplementary file 2. [file elife-48281-supp2.docx]

**Supplementary File 2**

**Pol II backtrack analysis**

|  | No. of backtracks per molecule | | Backtrack durations  (s) | | Backtrack Positions  (bp)^a^ | | | | | Backtrack depth (- bp)^b^ | |
| --- | --- | --- | --- | --- | --- | --- | --- | --- | --- | --- | --- |
|  | Mean  (sd)^c^ | Median | Mean  (sd) | Median | Mean  (sd) | Median | Before dyad (%) | After dyad (%) | N^d^ | Mean  (sd) | Median |
| xWT  Nucleosome | 5  (4.8) | 3.5 | 14.4  (18.7) | 6.1 | 54.4  (30.9) | 51.3 | 83 | 17 | 224 | 4.6  (2.8) | 4.3 |
| hWT  Nucleosome | 5.4  (4.7) | 4.5 | 19.1  (24.6) | 8.4 | 59.1  (33.7) | 51.1 | 69 | 31 | 242 | 5.4  (3.8) | 4.5 |
| H2A.Z  Nucleosome | 3.9  (3.6) | 3.5 | 23.2  (25.9) | 12.9 | 59.8  (36.7) | 58.0 | 66 | 34 | 172 | 4.5  (2.6) | 3.9 |
| uH2B  Nucleosome | 5.7  (5.3) | 4.0 | 16.9  (22.8) | 6.4 | 64.3  (34.9) | 59.6 | 68 | 32 | 285 | 4.8  (3.4) | 3.9 |
| Bare NPS  DNA^e^ | 0.2  (0.8) | 0.0 | 2.8  (1.8) | 2.5 | 62.5  (49.1) | 76.0 | 50 | 50 | 8 | 4.8  (3.4) | 3.2 |

1. Backtrack positions are normalized to the beginning of NPS, from 1 to 147 in bp. Only backtracks that occur along the NPS are analyzed. Although they are not numeric in nature, the median value estimates where backtracks most likely occur. Before and after dyad are percentage of backtracking events that occur before or after dyad (74 bp)
2. Backtrack depths are the number of basepairs (bps) Pol II backtracks from the 3’ end of mRNA, expressed in (- n) bp.
3. Values are expressed in mean and sd (standard deviation), with sd values in parenthesis.
4. N is total number of backtracks analyzed from all traces.
5. Because Pol II barely backtracks on bare NPS DNA (N = 8 for all traces), it might not be meaningful to comparable some parameters to those of nucleosome samples.
